# Cellular Phenotype Plasticity in Cancer Dormancy and Metastasis

**DOI:** 10.3389/fonc.2018.00505

**Published:** 2018-11-05

**Authors:** Xiao Yang, Xinhua Liang, Min Zheng, Yaling Tang

**Affiliations:** ^1^State Key Laboratory of Oral Diseases and National Clinical Research Center for Oral Diseases and Department of OralPathology, West China Hospital of Stomatology, Sichuan University, Chengdu, China; ^2^State Key Laboratory of Oral Diseases and National Clinical Research Center for Oral Diseases and Department of Oral and Maxillofacial Surgery, West China Hospital of Stomatology, Sichuan University, Chengdu, China; ^3^Department of Stomatology, Zhoushan Hospital, Wenzhou Medical University, Zhoushan, China

**Keywords:** cancer cell dormancy, EMT, MET, cellular phenotype plasticity, cancer metastasis

## Abstract

Cancer dormancy is a period of cancer progression in which residual tumor cells exist, but clinically remain asymptomatic for a long time, as well as resistant to conventional chemo- and radiotherapies. Cellular phenotype plasticity represents that cellular phenotype could convert between epithelial cells and cells with mesenchymal traits. Recently, this process has been shown to closely associate with tumor cell proliferation, cancer dormancy and metastasis. In this review, we have described different scenarios of how the transition from epithelial to mesenchymal morphology (EMT) and backwards (MET) are connected with the initiation of dormancy and reactivation of proliferation. These processes are fundamental for cancer cells to invade tissues and metastasize. Recognizing the mechanisms underlying the cellular phenotype plasticity as well as dormancy and targeting them is likely to increase the efficiency of traditional tumor treatment inhibiting tumor metastasis.

## Introduction

**Figure 1:**
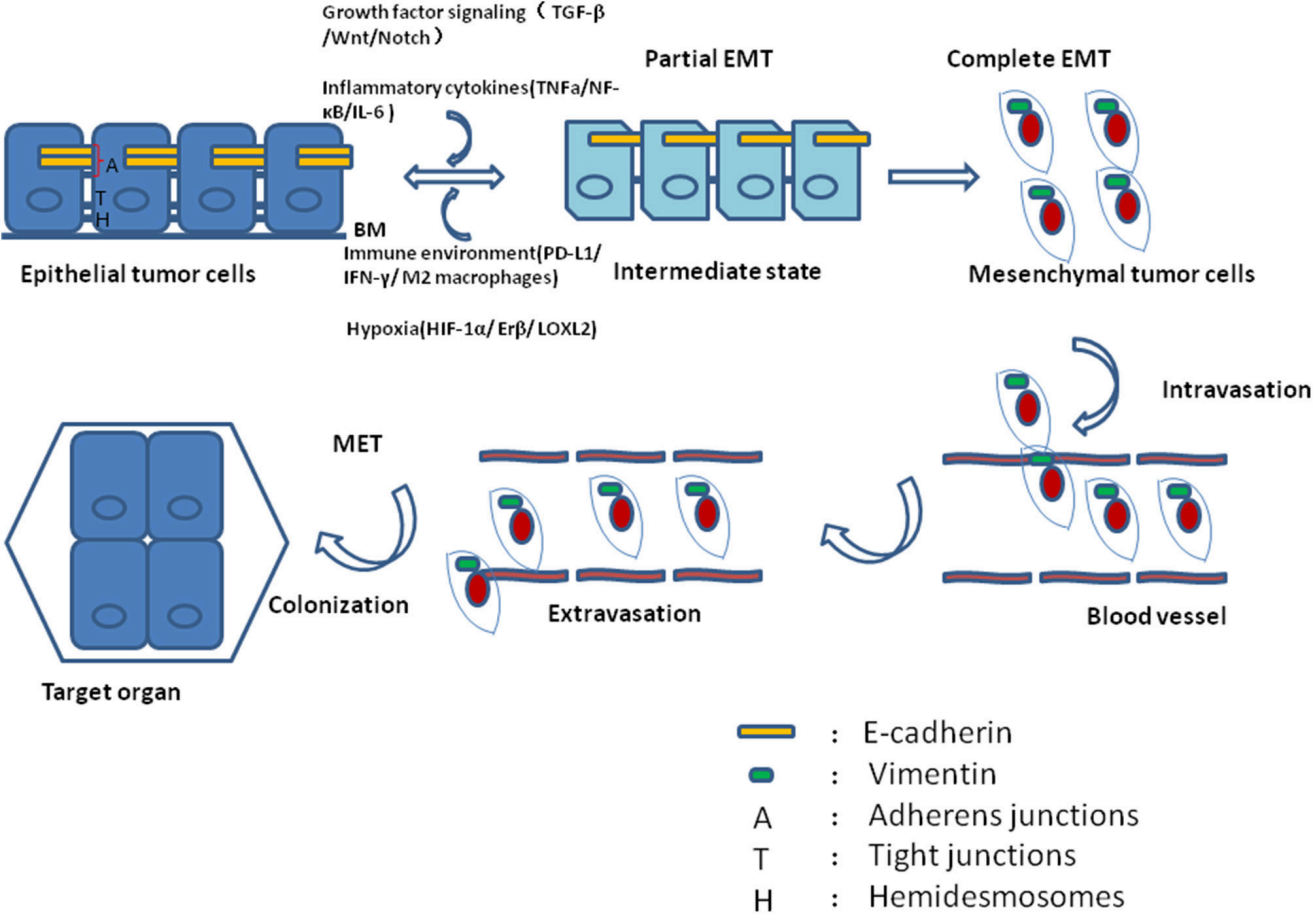
During tumor progression, epithelial tumor cells undergo partial EMT through their crosstalk with contextual signals, including growth factor signaling, inflammatory cytokines, immune environment and hypoxia. Concomitantly, these tumor cells within intermediate states gain the ability of motility while remaining cell-cell junction and expressing E-cadherin. After complete EMT, the intermediate tumor cells lose their epithelial junctions, their cytoskeleton reconstructed and vimentin filaments were generated instead of E-cadherin. After intravasation and extravasation through blood vessel, the EMT positive tumor cells arrive at their target organ, and they have to experience MET to accomplish colonization.

The cellular phenotype plasticity was first observed in developmental morphogenesis and then was shown to play a critical role in epithelium-derived carcinoma metastasis (1, 2). Epithelial cells are polygons, have apical-basal polarities, tight intercellular connections, and are fixed to the basement membrane via hemidesmosomes. E-cadherin is the gatekeeper to maintain the tight connections of epithelial cells and cytokeratin makes up the largest subgroup of intermediate filament proteins. In contrast, mesenchymal cells with spindle fibroblast-like morphology rarely build tight connections and in general have no E-cadherin, but Vimentin is the most abundant protein in cell skeleton (3). Epithelial mesenchymal transition (EMT) is a process of cell dedifferentiation and adaption of the cellular morphology and behavior. During EMT program, the polygonal cells turn into fusiform fibroblast-like cells, they lose their epithelial junctions, including hemidesmosomes, adherens junctions and tight junctions and reduce the apical-basal polarity. Their cytoskeleton reconstructed and vimentin filaments were generated instead of E-cadherin. These mesenchymal cells transformed from epithelial cells acquire the ability of migration and invasion. Of note, the process of mesenchymal cells transforming into epithelial cells is regarded as mesenchymal epithelial transition (MET) which is reversible from EMT (4). Activating EMT program is thought to be one of the driving forces to promote tumor metastasis in that EMT can induce migrating and invading, while MET enables tumor cells to colonize in the target organs. Recently, it was revealed that circulating tumor cells (CTCs) always display partial EMT activation with the properties of both epithelial and mesenchymal cells (5). Tumor cells within this intermediate state have the ability of motility while remaining cell-cell junction and express E-cadherin (6, 7) (Figure 1). During tumor progression, EMT is initiated by a number of contextual signals from tumor microenvironment. Many signals inducing EMT program tend to be cell or tissue type specific and may require cooperation between each other. The developmental signaling pathways, including TGF-β, Wnt, Notch, and growth factor receptor signaling cascades, and inflammatory cytokines, as well as hypoxia all have been shown to stimulate EMT (8, 9). In general, there are three core groups of transcription factors that orchestrate EMT program. The first one is Zeb, the distantly related zinc finger family, including Zeb1 and Zeb2, which are capable of repressing E-cadherin expression. The second group is Snail zinc finger family, including Snail1 and Snail2, both of which are able to combine with the promoter of E-cadherin to suppress its transcription (10). The third group of EMT transcription factors is the basic helix-loop-helix (bHLH) family, including Twist1, Twist2, and E12/E47 which could induce EMT program effectively (11). However, different EMT transcription factors have considerable variability and tissue specificity in driving it (12). Even the same EMT transcription factor may dictate distinct cellular responses in different tumor types (13). Moreover, recently EMT positive tumor cells have been shown to be related to low proliferation rate or quiescence and EMT may have a potential role in cancer cell dormancy (14, 15).

Many patients relapse or show recurrent disease and suffer from metastases several years even decades after they have had radical surgery (16). This phenomenon can be interpreted as cancer dormancy, a period in cancer progression in which residual diseases exist but remain clinically asymptomatic for a certain time. It has been demonstrated that cancer dormancy represents a stage of growth arrest, which may appear in the course of primary tumor formation, after dissemination of the primary tumor cells or in micrometastasis (17). However, cells of primary tumor, and metastasis may enter into dormancy through different inductions or scenarios (18): the early dormancy may take place at the incipient stage of primary tumor, as neoplastic cells obtained some somatic mutations that were required for resisting apoptosis or senescence as well as escaping immunosurveillance. (19, 20). In addition, circulating tumor cells (CTCs) or cells seeded in the pre-metastatic sites may undergo dormancy because they are not adapted to the foreign microenvironments (17). Of note, micrometastasis may enter a state of dormancy possibly because the division and apoptosis of its constituent cells are in equilibrium, and the volume of metastatic tumor does not obviously expand (17). Alternatively, tumor cells may display a very low proliferation rate because of counterbalance between increases in the number of tumor cells and immune attacks (16). It was demonstrated that cancer cell dormancy is one of the major causes of metastasis which is characterized by non-dividing cells, arrested in G0-G1 (21, 22). Moreover, it was shown that tumor cells with the traits of invasiveness and stemness which have undergone EMT program always manifested features of dormancy, including low levels of Ki67 (23).

Current research shed light on the molecular circadian mechanisms and changes of cellular behaviors during cancer dormancy. Several innovative experimental methods, mouse models as well as mathematical models have been used to explore cellular phenotype plasticity and cancer dormancy (24). In this review, the cellular phenotype plasticity and its relationship with cancer dormancy and metastasis are discussed, with an emphasis on the cross talk between EMT, MET, dormant cancer cells, and their microenvironments. In addition, the role of metabolism in cancer dormancy is also included.

## EMT and cancer dormancy

Tumor cells that have undergone EMT acquired the capacity of migration and invasion that enable them to separate from primary tumor, enter into the circulation, and colonize the target organ. Concomitantly, tumor cells that initiate metastasis seem to be cancer stem cells (CSCs) or, at least, have some characteristics of these cells. Although there has been evidence for a causal relationship between CSCs and EMT process (25–28), it remains elusive which cellular type, mesenchymal or epithelial, could endow tumor cells with stem cell properties (29, 30). Experimental researches as well as mathematical models indicate at the existence of an intermediate relatively stable state in which tumor cells have both mesenchymal and epithelial features (31, 32). Cells residing in hybrid EMT state can maintain contacts, but gain a migratory phenotype that allows them to migrate in small groups, termed “collective migration” (33). Furthermore, this hybrid EMT state endows tumor cells with stemness, while a fixed cellular phenotype does not (34). Schmidt and coworkers showed that transient activation of Twist produces a cell subpopulation with characteristics of both epithelial and mesenchymal, which displays the highest stemness capacity (35). In ovarian cancer, tumor cells in the intermediate EMT state display highest sphere-forming ability, which are also indicative for a worse prognosis in patients (36). It was hypothesized that CSCs contribute to tumor recurrence and metastasis as they have the features of invasiveness, stemness, drug resistance, and the capacity to form metastases at target organ (37–39).

However, Zheng et al. reported that tumor cells could invade and metastasize without undergoing EMT process. They generated genetically engineered mouse models for pancreatic ductal adenocarcinoma with deletion of Snail or Twist, which showed a reduced expression of the mesenchymal marker a-smooth muscle actin (aSMA), and they observed that EMT suppression did not alter systemic dissemination, invasion, and metastasis. Therefore, they concluded that EMT process is dispensable for metastasis in pancreatic cancer (13). However, Aiello and his colleagues proposed that in this genetically engineered mouse model, aSMA is rarely expressed during activation of EMT (40). Moreover, primary tumors described by Zheng et al. continued to express considerable levels of Zeb1, Sox4, and Slug, after deletion of Snail or Twist1. Quantitative PCR of cells isolated from primary tumor from knockout mice showed that mRNA levels of Zeb1 and Slug were decreased by 2-fold which is seemingly inconsequential functionally. Similarly, Fischer et al. recently reported that EMT program is not required for breast-to-lung metastasis (41). They used a mesenchymal-specific Cre-mediated fluorescent marker switch system mouse model to track tumor cells that have undergone EMT process. They used a Fsp1-cre transgene and described Fsp1 as a pivotal gatekeeping gene of EMT program as it was shown in another publication that Fsp1 was required for renal proximal tubular epithelial cells to undergo EMT *in vitro* (42). However, according to various studies about EMT in many kinds of tumors, it is irrational to consider a marker of EMT in renal tubular cells as a useful indicator in breast cancer epithelial cells (43, 44). Österreicher and co-workers demonstrated that Fsp1 was not an integral component of EMT process (45). Ye et al. proposed that Fsp1 was expressed in a very small part of breast cancer cells that have been induced EMT by Snail and Zeb1, the master regulators of EMT (46). So the illustration that EMT program is not involved in the invasion-metastasis cascade is not sufficient for ruling out EMT contribution in metastases.

**Figure 2:**
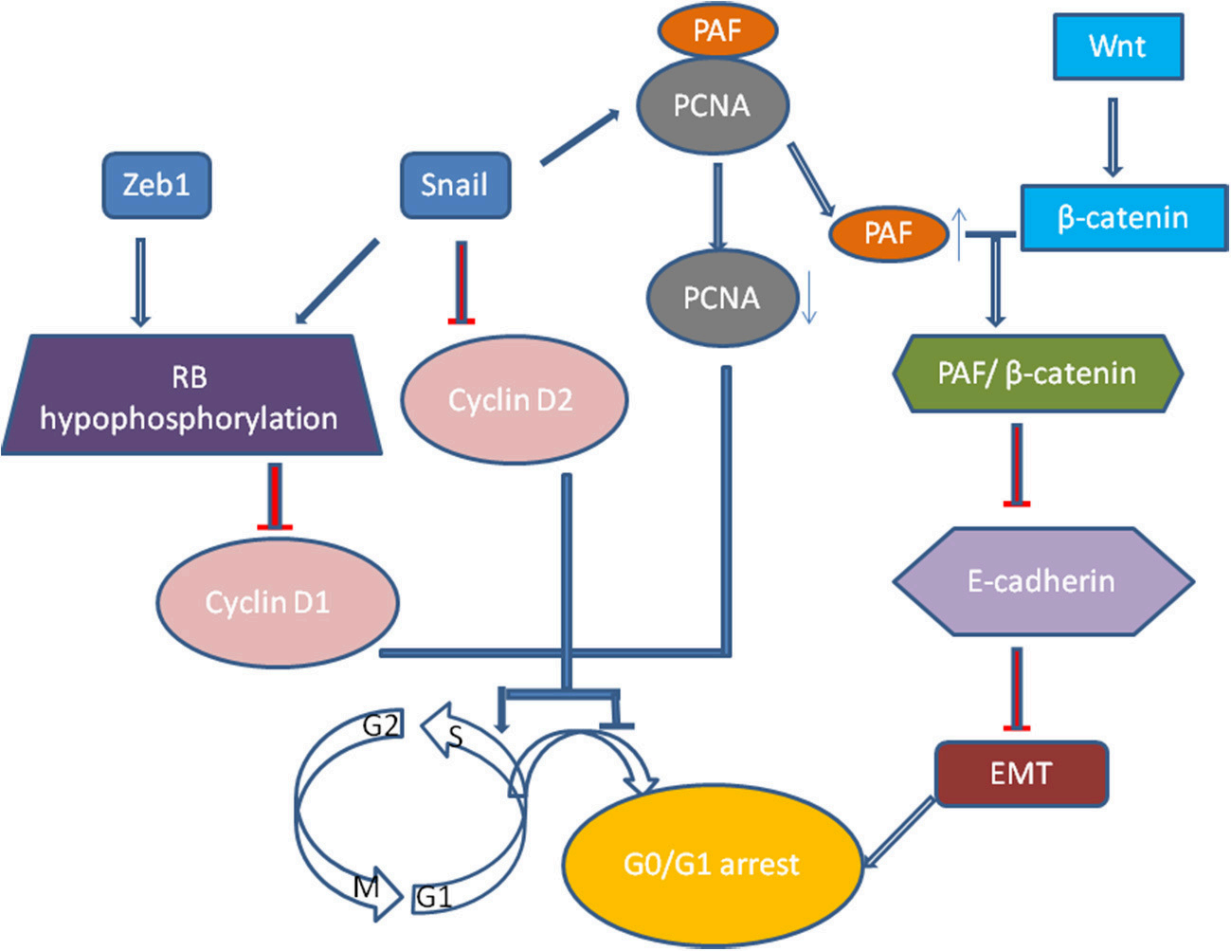
Snail, one of EMT transcription factors, could dramatically impair cell-cycle progression by repressing the transcription of cyclin D2. Moreover, Snail could suppress tumor cell proliferation through binding to flanking region of proliferating cell nuclear antigen (PCNA) gene to decrease its expression. When Snail suppresses the expression of PCNA, the PAF (PCNA-associated factor) then dissociates from PCNA complexes and combines with β-catenin, which then upregulates Wnt/β-catenin target gene expression via activating Wnt signaling pathway and represses E-cadherin expression. And Zeb1 and Snail induce G1 arrest by promoting hypophosphorylation of Rb protein, the suppressor protein of tumor progression, and decreasing the expression of cyclin D1.

Recently, growth arrest or cell quiescence has been shown to be an attribute of CSCs and disseminated tumor cells (DTCs) which are thought to be dormant and persisting cancer cells. It was well realized that TGF-β induced EMT process is related to low proliferation rate and cell arrest in epithelial cells (47). Vega et al. illustrated that Snail, one of EMT transcription factors, could dramatically impair cell-cycle progression by repressing the transcription of cyclin D2 whose activity was required for cell division (48). Then, it was extended that Snail could suppress tumor cell proliferation through binding to flanking region of proliferating cell nuclear antigen (PCNA) gene to decrease its expression (49). PCNA plays a vital role in DNA replication as it serves as an auxiliary portion of the DNA polymerase-δ complex and also has some common properties with cyclin (50). When Snail suppresses the expression of PCNA, the PAF (PCNA-associated factor) then dissociates from PCNA complexes and combines with β-catenin, which then upregulates Wnt/β-catenin target gene expression via activating Wnt signaling pathway and represses E-cadherin expression (51). And Zeb1 and Snail induce G1 arrest by promoting hypophosphorylation of retinoblastoma (Rb) protein, the suppressor protein of tumor progression, and decreasing the expression of cyclin D1(52) (Figure 2). The proliferation of tumor cells of squamous cell carcinoma metastases are suppressed by activating Twist1 (53). Thus we proposed that quiescence is a general characteristic of tumor cells that have undergone EMT (52). However, how these tumor cells remain and exit dormancy has not been verified, and the dynamic changes of the cellular phenotype in tumor progression have not been precisely shown *in vivo* (54, 55).

## Cellular phenotype plasticity in primary and metastatic dormancy

**Figure 3:**
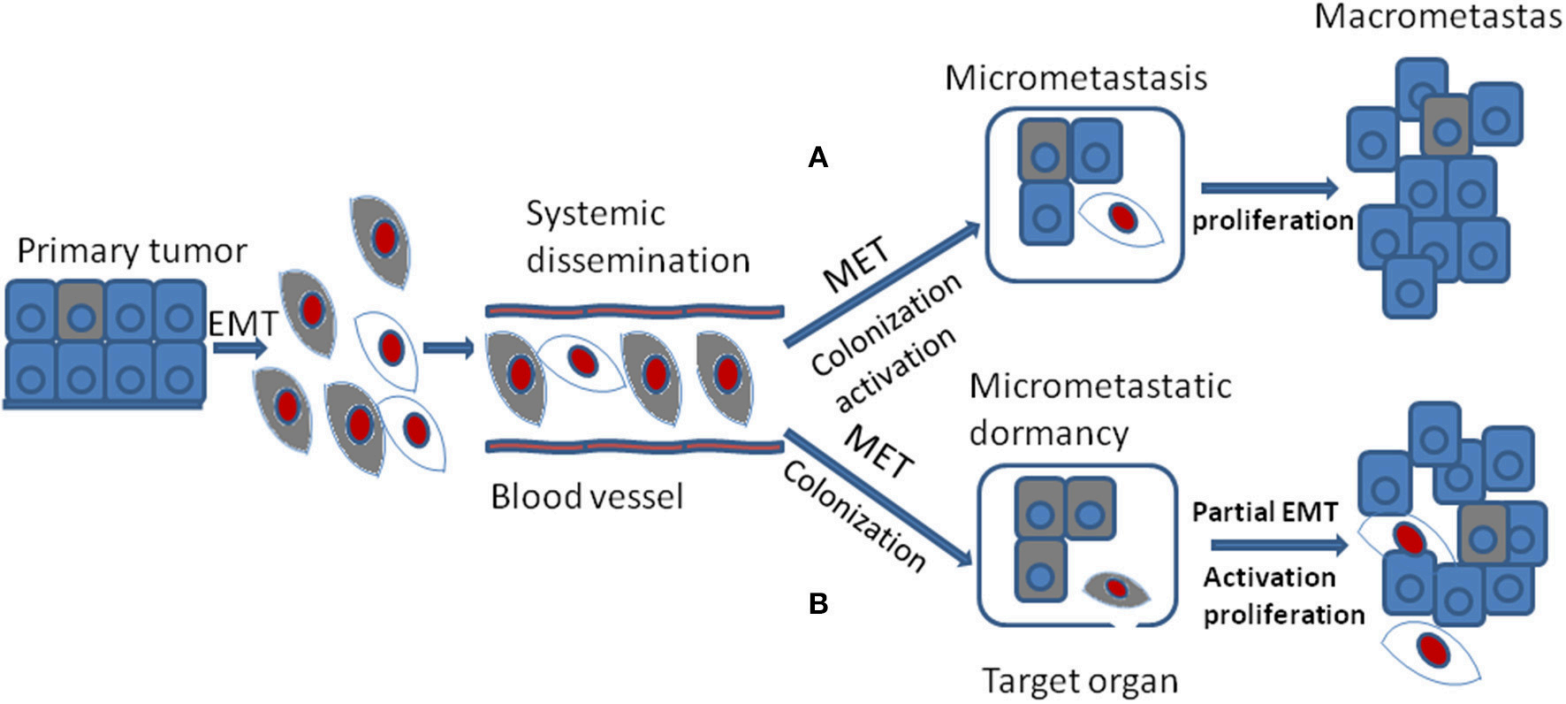
During tumor progression, EMT positive tumor cells from primary tumor gain the ability of migration and invasion and their proliferation is inhibited which are thought to be dormant tumor cells. They are colored gray. After systemic dissemination, only a small portion of circulating tumor cells arrive at their target organ and they have to undergo MET to accomplish colonization. Until now, there are two different viewpoints concerning the following stages. **(A)** when EMT positive tumor cells arriving at target organ, the MET program induce their colonization and activation. The activated tumor cells then proliferate into macrometastasis. They are colored red. **(B)** when EMT positive tumor cells arriving at target organ, the MET program may just promote their colonization or formation of micrometastasis that stay in dormant stage. Then the secondary partial EMT is thought to induce their activation and turn the dormant micrometastasis into macrometastasis.

In spite of the fact that the DTCs reveal lots of morphological and molecular characteristics of EMT, cells of metastases in target organ or tissue always display epithelial traits which contradicts with the hypothesis that EMT positive tumor cells should generate metastases with mesenchymal phenotype(56). The MET program has been proposed to account for the above discrepancy: EMT positive tumor cells acquire the ability of migration and invasion and thus separated from primary tumor; once arriving at target sites, mesenchymal tumor cells need to revert to epithelial phenotype to complete colonization and metastasis initiation (Figure 3A). It was shown that the expression of E-cadherin is regulated by epigenetic mechanisms, especially methylation of its promoter. Of note, aberrant hypermethylation of E-cadherin promoter region 5' CpG island repress E-cadherin expression and loss of methylation upregulate E-cadherin expression (57). It has been shown that the corresponding metastasis reveals increased E-cadherin expression compared with primary tumor in breast cancer. Additionally, injecting mesenchymal MDA-MB-231 cells via tail vein into secondary organ lead to re-expression of E-cadherin through passive loss of methylation process in its promoter, and the consequent MET (58). Bonnomet et al. demonstrated that the primary MDA-MB-468 xenografts reveal heterogeneous expression of Vimentin compared to lung metastases in which high levels of Vimentin and Snail were detected in EMT-induced CTCs. They proposed that the Vimentin-positive CTCs need to reverse EMT to facilitate metastatic growth (59). Furthermore, suppression of Prrx1, another inducer of EMT, was essential for lung metastases in human breast cancer (60). Consistent with these studies, in a spontaneous squamous cell carcinoma mouse model, inducing EMT via Twist1 activation promoted tumor progression, including invasion, intravasation, and extravasation, while turning off Twist1 in metastatic foci to induce MET was required for macrometastases formation (53). Together, all the above confirm that MET is required for metastases colonization. However, this has not been verified *in vivo* (53).

The occurrence of cellular phenotype plasticity in tumor metastases *in vivo* has been under intense debate (61, 62). Giancotti put forward two kinds of hypotheses about the relationship between EMT, MET, and metastases (17). First, EMT positive CSCs with self-renewal capacity cycle slowly in the metastatic foci and they could generate their immediate progeny that re-expresses E-cadherin and up-regulates proliferation capacity to spur metastatic outgrowth. Second, EMT positive CSCs may enter into dormant state and they need to undergo MET process to regain the ability of proliferation.

Another standpoint about the relationship between EMT, MET, and metastases is quite different. Some researchers hold the view that EMT may promote dissemination, invasion, extravasation, and the dormant state of CTCs or DTCs in primary tumors. And MET may be critical for colonization, ectopic survival as well as micrometastatic dormancy, once the metastasis in distant place is involved. Furthermore, a secondary partial EMT is supposed to be the foundation of these silent microscopic metastases outgrowth into macroscopic metastases (63) (Figure 3B).

In line with this standpoint, present evidences showed that the MET program in metastatic target organs could rebuild the connection between tumor cells and may induce dormancy, thus, the dormant tumor cells may have the ability to stay alive at a lower metastatic load in the inhospitable metastatic microenvirenment (15, 64). Inflammation has been shown to be one of the microenvironment factors that influence cellular phenotype plasticity. By the co-culture system of macrophages with epithelial and mesenchymal cell lines, Yang et al. proposed that in liver metastasis of breast cancer, M1 macrophages may induce MET and thus contribute to dormant behaviors in tumor cells, while EMT program regulated by M2 macrophages might drive the outgrowth of metastasis (65). In an *in vivo* mouse model of dormant lung metastasis, local inflammation induced by lipopolysaccharide (LPS) drove the awakening of these latent cells which then developed into macrometastasis in the lung parenchyma. The above described process of inflammation activating dormancy depended on Zeb1 and induction of EMT program via Zeb1 expression was sufficient to significantly increase the number and volume of metastases (66).

Additionally, Chao and his colleagues found that hepatocyte coculture triggered the re-expression of E-cadherin in breast and prostate cancer cells imparted by MET program. Activation of MET program then increased the survival and chemoresistance of metastatic tumor cells which is a characteristic of tumor dormancy (67). The hepatic non-parenchymal cells in metastatic hepatic niche can induce EMT by EGFR partly, which induced dormancy to activate and initiate metastasis (68). In breast cancer, by designing several breast cancer cell lines with firefly luciferase in the lungs of mice, Wendt et al. proposed that EMT and its subsequent reduction of E-cadherin circumvent metastatic dormancy through promoting integrin β1 expression which is essential for metastatic outgrowth (63). In addition, they also found that dormant D2.OR cells are E-cadherin high indicating these cells have underwent MET, and the expression of Twist activated metastatic outgrowth in these dormant cells. The switch from quiescence to activation of dormant metastatic tumor cells depended on fibronectin production and signals from integrin β1, leading to cytoskeletal reorganization (69). Additionally, activation of EMT often induced the expression of fibronectin and the subsequent changes in cytoskeletal architecture, suggesting that EMT program is responsible for the dormancy-to-activation switch in metastases. The integrinβ1- focal adhesion kinase (FAK) signaling axis, an important regulator of EMT process in breast cancer cells, played a critical role in facilitating proliferation of micrometastatic cancer cells (69–72). By modeling the dynamic process of EMT, MacLean et al. proposed that EMT occurred in metastasis and provide a plausible mechanism by which macroscopic metastasis could arise from dormant micrometastasis (24).

These above findings show that MET is required for metastases colonization and micrometastatic dormancy, and partial EMT is induced for the activation of these dormant tumor cells and their outgrowth into macroscopic metastases (15, 65). These studies indicate that cellular phenotype plasticity plays an important role in dormant-to-proliferative metastases transition.

## Microenvironment with EMT and dormancy

Both genetic and epigenetic mechanisms have been shown to contribute to cellular phenotype plasticity during tumor progression (73). Though the genetic factors have been extensively discussed, epigenetic mechanisms have not been fully understood, and cellular phenotype plasticity represents an important example of how epigenetic factors promote tumor progression. EMT process is regulated at the transcriptional and translational level by a variety of pro-invasion signals from microenvironment, such as growth factor signaling, hypoxia, nutrient conditions and inflammatory cytokines (Table 1). Additionally, tumor cells can also enter into dormant stage through their crosstalk with microenvironment to escape immune surveillance, apoptosis and senescence. Hence, extracellular signals from tumor microenvironment play a vital role in inducing tumor dormancy as well as activating EMT program.

**Table 1:** Molecule mechanism of EMT and cancer dormancy within microenvironment.

**Growth factor signaling**	**Inflammatory cytokines**	**Immune environment**	**Hypoxia**
TGF-β	TNFa	PD-L1	HIF-1α
BMP	NF-κB	IFN-γ	ERβ
Wnt	IKK-β	IDO-Kyn-AhR-p27	LOXL2
Notch	IL-6	M2 macrophages	LIFR: STAT3: SOCS3
Hedgehog and receptor tyrosine kinases	IL-1β		uPAR
Wnt/β-catenin/LEF-1	VCAM-1	
PTKs	integrin α4β1	
[ERK/p38]^low^		
P27		

*^†^TGF-β, transforming growth factor-β family; BMP, bone morphogenetic protein; PTKs, receptor tyrosine kinases*;

*^‡^VCAM-1, vascular cell adhesion molecule 1;^§^PD-L1, programmed cell death-Ligand 1; IFN-γ, interferon-γ;^¶^HIF-1α, Hypoxia-inducible factor-1α; ERβ, estrogen receptor β; uPAR, urokinase receptor*.

### Growth factor signaling

**Figure 4:**
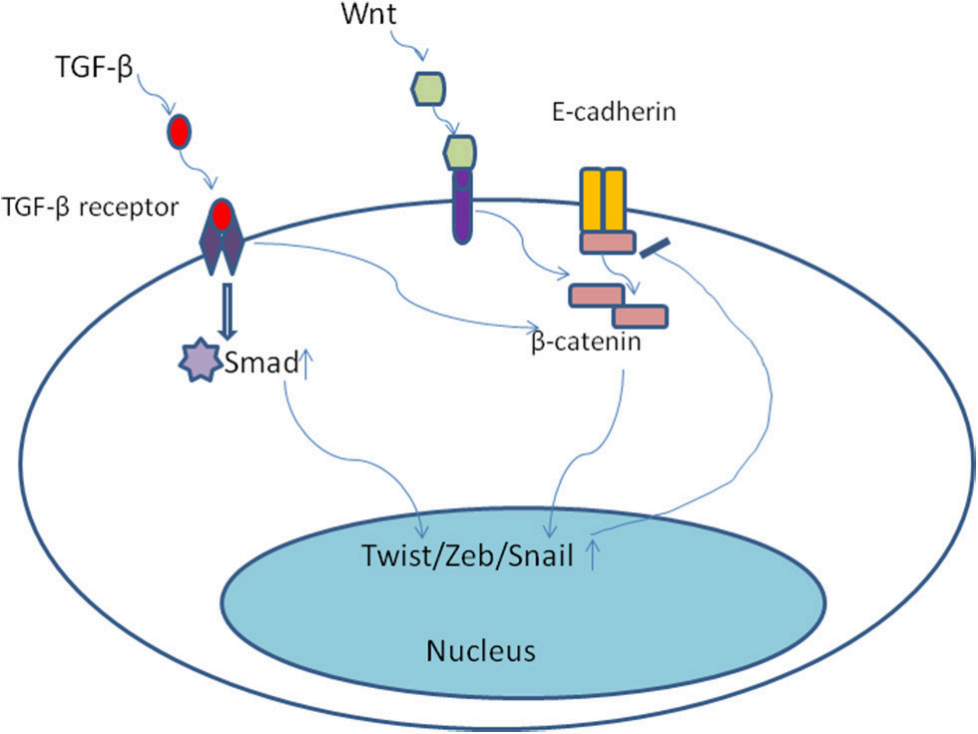
Once activated, TGF-β bound to its cell surface receptor forming tetrameric complexes to promote the formation of Smad family transcription factors, which then move into nucleus and combine as well as concomitantly up-regulate the expression of other transcription factors including Snail, Zeb, and Twist. Moreover, TGF-β/Smad involved in EMT program need the cooperation of some other pathways, including Wnt/β-catenin. TGF-β could up-regulate the expression of β-catenin and its nuclear accumulation.

The developmental signaling pathways orchestrate EMT program including transforming growth factor-β family (TGF-β), bone morphogenetic protein(BMP), Wnt, Notch, Hedgehog, and receptor tyrosine kinases (74). Most notably, TGF-β, mainly derived from stromal fibroblasts in tumor microenvironment (75), seems to be a primary driver of EMT which has a fundamental role in tumor progression (8). In the late-stage of tumor progression, TGF-β signaling pathway appears to be redirected away from inhibiting tumor cells proliferation and is thought instead to induce EMT program. Of note, the EMT positive tumor cells always display distinctive cellular characteristics, including invasiveness, stemness, and the ability of forming metastases (75). Once activated, TGF-β bound to its cell surface receptor forming tetrameric complexes to promote the formation of Smad family transcription factors, which then move into nucleus and combine as well as concomitantly up-regulate the expression of other transcription factors including Snail, Zeb, and Twist (76, 77). Moreover, TGF-β/Smad involved in EMT program need the cooperation of some other pathways, including Wnt/β-catenin/LEF-1 (78) and Ras kinase cascade by activating receptor tyrosine kinases (PTKs) (79). For example, TGF-β could up-regulate the expression of β-catenin and its nuclear accumulation (80) (Figure 4). Malanchi and coworkers demonstrated that TGF-β 3 produced by DTCs could induce periostin production, which recruits Wnt ligands, and triggers Wnt signaling pathway in breast cancer cells (81). Additionally, Steinway and coworkers found that Wnt and Sonic hedgehog signaling was activated by the constitutive presence of TGF-β during EMT program by using network modeling in hepatocellular carcinoma (82). Their interactions then bring about EMT and contribute to invasion-metastasis cascade. TGF-β signaling has been shown to result in epigenetic regulation of downstream genes. For example, Smad2 and Smad3 could combine with epigenetic regulators, which replace repressive histone modifications and create a poised chromatin structure (83). Notably, transforming growth factor-β2 (TGF-β2) and TGFβ-receptor-III (TGF-β RIII) signaling in bone marrow could induce dormant stage of DTCs via activating P38a/β, driving a [ERK/p38]^low^ signaling ratio. And TGF-β2 induced dormancy still need the cooperation of P27 which is activated by TGFβ-receptor-I (TGF-β RI), TGF-β RIII and SMAD1/5 (84). On the contrary, low levels of TGF-β2 in lungs changed dormant CTCs behaviors and followed by outgrowth of metastases (85). These findings suggest that TGF-β from tumor microenvironment could mediate EMT as well as induce tumor cells dormancy via different molecular mechanisms.

### Inflammatory cytokines

Inflammatory microenvironment has proven to play an important role in both EMT program and tumor dormancy. Wu and his colleagues found that the function of Snail corresponded with the activation of NF-κB and the effect of inflammation cytokines was repressed by turning off Snail expression, thus they proposed that the stabilization of Snail could be maintained by inflammation cytokine TNFa through NF-κB pathway (86). TNFα could up-regulate the expression of Twist1 through activating IKK-β and NF-κB p65 (87). Indeed, IL-6 could promote EMT process in breast cancer cells (9). Nitric oxide produced by fibroblasts regulates IL-1β and NF-κB dependent chemo-resistance which is one of the characteristics of dormancy in pancreatic tumor cells, while inhibiting NF-κB re-sensitizes these tumor cells to chemotherapy induced apoptosis (88). However, Lu and co-workers characterized a bone metastasis dormancy model of breast cancer and showed that the expression of vascular cell adhesion molecule 1 (VCAM-1) was in part mediated by NF-κB pathway (89). VCAM-1 recruited mononuclear osteoclast precursors and elevated their activity by interacting with integrin α4β1, the cognate receptor of VCAM-1. The recruitment and activation of osteoclasts will in turn facilitate tumor cell proliferation and foster micrometastatic expansion (90). Inhibiting the expression of VCAM-1 and integrin α4β1 would effectively suppress bone metastasis progression of breast cancer. Macrophages represent the key cellular component of inflammation and their density may influence the prognosis of cancer patients (65, 91). M2 macrophages promote EMT program and the subsequent dissemination from primary tumor as well as the formation of distant metastasis (92, 93). In addition, in regard to the influence of inflammation on tumor dormancy, De Cock and coworkers showed that inflammatory environment in lung metastasis awakened previous latent cells which developed into macrometastasis through inducing expression of Zeb1 (66). It was also proposed that in primary site, chronic inflammation was a major factor in activating dormant tumor cells and leading to the formation of primary tumor as well as distant pre-metastatic microenvironment (94).

### Immune environment

Apart from soluble ligands, the immunesystem is one of the key components within the microenvironment (95, 96). Immune evasion is required for tumor development and is characterized by non-effective anti-tumor immunoreactions and increase of immune suppressors. Lou and his colleagues found a strong association between EMT and inflammatory tumor microenvironment. In addition, they demonstrated that EMT signature was associated with up-regulation of immune suppressive receptors and ligands, including PD-L1, PD-L2, PD-1, TIM-3, B7-H3, BTLA, and CTLA-4 in lung adenocarcinomas. Their results demonstrated a previously unrecognized relationship between EMT and immune evasion in lung cancers (97). Alsuliman et al. recently reported a bidirectional effect between EMT program and PD-L1 mediated immune escape in that activation of EMT up-regulated PD-L1 expression and suppression of PD-L1 down-regulated the mesenchymal phenotype in breast tumor cells (98). Additionally, EMT transcription factors, such as Snail and Zeb, have been linked with immune inhibition in tumor (99). For instance, it was reported that microRNA-200/Zeb1 axis could regulate PD-L1 expression even in the absence of IFN-γ in lung tumor cells and tumor cells with mesenchymal phenotype are intrinsically capable of immunosuppressing (100). In addition, Snail-activated EMT process could induce regulatory T cells and impaired dendritic cells in murine and human melanoma cells (101). It was well known that tumor cells could escape the killing by cytotoxic immune cells via entering into dormancy. Immunologic dormancy was first observed in post-transplantation patients clinically. According to Robert Schreiber and his colleagues, tumor mass dormancy is achieved by the equilibrium of tumor growth and immune-mediated tumor killing (102). While, tumor cell dormancy represents a process of immune factor-mediated cell cycle arrest at the cellular level (103). Even though, tumor cells could escape immune surveillance via entering into dormant state, it is possible that anti-tumor immunity generate certain mechanisms to render them dormant (104, 105). IFN-γ, one of the pleiotropic cytokines, is a vital anti-tumor immune factor mainly produced by cytotoxic CTLs and NK cells. By binding to its receptor, IFN-γ triggers the expression of many interferon induced genes that could cause growth arrest or cell death (106). Similarly to the function of EMT, IFN-γ could also inhibit immune response by up-regulating the expression of immunosuppressive molecules such as PD-L1, thus suppressing immune elimination of tumor cells (107). Müller-Hermelink et al. demonstrated that IFN-γ was implicated in tumor equilibrium as tumorous growth and expansion are held in check by immune response (105), suggesting that IFN-γ may be involved in tumor dormancy. Recently, Liu et al. found that IFN-γ induced tumor-repopulating cells (TRCs) to enter into dormancy through triggering the IDO-Kyn-AhR-p27 cascade (108). From the above researches, we proposed that the immune-suppression induced by EMT program may also facilitate tumor cell dormancy.

### Hypoxia

Hypoxia is related to metastases and poor prognosis in various types of human cancers (109, 110). Hypoxia-inducible factor-1α (HIF-1α) is the main factor mediating cellular hypoxic response (111). HIF-1α is ubiquitinated and degraded under abundant oxygen environment, while hypoxia enables HIF-1α translocation to the nucleus and eventually promotes the adaption of tumor cells to hypoxic microenvironment which often occurs in a wide range of cancers (112). Intratumoural hypoxia could induce EMT program in tumor progression, the one important signaling pathway involves HIF-1α and Twist in response to hypoxia (113). Mark et al. also showed that TGF-β and hypoxia decrease ERβ expression to promote EMT and the underlying mechanism involves ERβ-mediated instability of HIF-1α and repression of VEGF-A which promotes the expression of Snail1 (114). So the hypoxia-associated environment is hospitable for tumor cells dissemination from primary site via activating EMT process, which is also responsible for the tolerance of traditional therapy as a result of dormant stage of tumor cells (115).

However, there are still studies contradicting with the above conclusions. Lysyl Oxidase Like 2 (LOXL2), one of the lysyl oxidase (LOX) family members and a matrix remodeling enzyme, promotes ECM remodeling (116), triggers EMT program (117, 118) and contributes to tumor progression and metastases. Weidenfeld and his colleagues recently demonstrated that conditional hypoxic environment triggered endogenous LOXL2 expression in MCF-7 cells and induced EMT and the acquisition of stemness features which eventually activated the transition of dormant tumor cells to metastatic growth (119). Moreover, IL-6 cytokine leukemia inhibitory factor (LIF) was hypothesized to facilitate breast cancer dormancy in the bone. Hypoxia could decrease the LIFR/STAT3/SOCS3 signaling pathway that consequently enables dormant tumor cells to escape dormancy or quiescence in breast cancer (120). Similarly, urokinase receptor (uPAR), a GPI-anchored membrane protein, mediates protease activity at the cell surface and initiate cell-signaling within the cell. Hypoxia, which is part of tumor microenvironment milieu, further up-regulated uPAR expression. Cell-signaling triggered by uPAR then promotes tumor cells EMT, migration, invasion, and escape from dormancy (121).

Alternatively, some researchers contradicted that cancer hypoxic microenvironment may inhibit invasion and induce dormancy (122, 123). It was also suggested that hypoxia induced “stress microenvironments” and might produce a dormant signature prime DTCs to enter into dormancy (124). Still further research is required to validate the influence of hypoxia.

## Therapeutic implications and perspectives

Cancer dormancy and cellular phenotype plasticity have come into sharp focus in recent years because of their close relationships with tumor metastases and relapses. In this review, we described different scenarios of how the transition from epithelial to mesenchymal morphology (EMT) and backwards (MET) were connected with the initiation of dormancy and reactivation of proliferation. Additionally, we enumerated the mechanisms underlying the plasticity as well as dormancy, which is helpful to provide targets to inhibit cancer metastases or relapses.

EMT program occurring in primary tumor endow tumor cells the abilities of dormancy, migration, invasion and stemness, which have a decisive role in the formation of metastases. Cancer cells with stemness tend to be more resistant to radiation and chemotherapy, which are challenging for traditional treatment (125). EMT process and the concomitant dormant DTCs and CTCs greatly complicate therapeutic methods, and targeting EMT program represents a crucial therapeutic strategy for cancer therapy. The more effective methods seem to inhibit the activation or functional consequence of EMT (73). For instance, rapamycin, along with 17-AAG and LY294002 could inhibit EMT via modulating TGFβ signaling pathway (126). By establishing a novel screening assay for inhibitors of EMT, Chua et al. found that ALK5, MEK, and SRC were potent repressors of EMT by interfering with EGF, HGF, and IGF-1 induced signaling (127). Additionally, Salinomycin has been identified as an agent that reduces epithelial differentiation and the proportion of CSCs (128). Pattabiraman and coworkers proposed therapy in breast cancers, which reversed EMT and induced MET by activating PKA and downstream epigenetic reprogramming (129). Yingling et al. demonstrated that a small molecule inhibitor of TGFβ receptor I, galunisertib, could reverse TGF-β mediated immune-suppression, EMT and tumor dormancy (130). Yadav and coworkers proposed that combination therapy targeting stem cell pathways including Wnt and Notch together with canonical oncogenic pathway could be effective (131). Given the discoveries that EMT program are induced by contextual signals, the combinatorial use of drugs that alter the proportion of stromal cells which is the source of TGFβ signaling in tumor microenvironment might further increase the effect of tumor treatment (132). However, current forms of therapies targeting core transcription factors of EMT remain technically challenging. The possible reason might be that some transcription factors do not bear druggable catalytic clefts (133).

Cancer cell dormancy provided new insights for cancer therapy and arise another complexity of therapeutic strategy (134). Neophytou and coworkers proposed that in breast cancer, the ultimate goal would be, if not to eliminate dormant metastatic cells, to prolong their dormant period (135). Thus, the purpose of treatmet might try to maintain the dormant stage of tumor cells or awaken them and eventually eliminate dormant cells. For instance, in head and neck squamous cell carcinoma, LB1, an inhibitor of protein phosphatase 2A can eliminate dormant tumor cells by promoting their activation from quiescence and increasing their sensibility to radiation or chemotherapy (136). It was also suggested that the Hippo pathway on tumor therapy contributes to the initiation and then stabilization of tumor dormant state (137). However, we should also take into account the possibility of causing extensive tumor recurrence through awakening dormant tumor cells that might cause great damage to patients. In addition, tumor angiogenic dormancy is maintained by the counterbalance between cancer cell proliferation and cancer cell apoptosis because of poor vascularization (138). It was proposed that metronomic chemotherapy is able to decline pro-angiogenic factors such as vascular endothelial growth factor (VEGF) and, simultaneously, increase anti-angiogenic factors such as thrombospondin-1 (TSP-1), probably playing a critical role in maintaining and inducing angiogenic tumor dormancy (139). However, because of the side effects of anti-angiogenesis, such as coagulopathy and gastrointestinal perforation, there is still much work to be done.

Recent findings have highlighted the essential roles of cellular phenotype plasticity in tumor progression and metastasis and its contribution to tumor dormancy. Nonetheless, we still lack a systematic framework of the whole process of cell states conversion and their connections with dormancy. Theoretically, these following unresolved issues seem worthy of further exploration: What promotes cellular dormancy, the fully epithelial, fully mesenchymal or intermediate state during tumor progression? Is MET essential for cells escaping from dormancy or tumor cells need to undergo partial EMT to outgrow into macrometastases? Which responsible extracellular signals and intracellular reaction pathways constitute the control circuits during the multiple alternative phenotypic states? Which genetic alterations maintain the different cellular phenotypes during dormant state? Moreover, most of the present data on cellular phenotype plasticity and tumor dormancy are actually derived from *in vitro* studies. Therefore, we need a number of sophisticated animal models for lineage tracing the tumor cells that have undergone EMT program in order to provide more preclinical evidences. In addition, multidisciplinary approaches like computational biology and experimental biology are needed to achieve a quantitative understanding of the relationship of EMT, tumor dormancy and metastasis in the future (5).

## Author contributions

All authors listed have made a substantial, direct and intellectual contribution to the work, and approved it for publication.

### Conflict of interest statement

The authors declare that the research was conducted in the absence of any commercial or financial relationships that could be construed as a potential conflict of interest.
